# Editorial: Neurobiology of food addiction

**DOI:** 10.3389/fnbeh.2023.1285557

**Published:** 2023-11-06

**Authors:** Carine Lampert, Marta G. Novelle

**Affiliations:** ^1^Department of Neurobiology, The University of Alabama at Birmingham, Birmingham, AL, United States; ^2^Department of Genetics, Physiology, and Microbiology (Unit of Animal Physiology), Faculty of Biology, Complutense University, Madrid, Spain

**Keywords:** food addiction, addictive behaviors, eating disorders, reward system, non-substance addiction

Food addiction (FA) is an intriguing issue that has received significant attention in recent years. The concept of “food addiction,” which refers to food as an addictive-potential factor, was first described in Randolph (1956). However, in the last two decades, this topic has been revisited and experienced a surge of academic interest as evidenced by numerous studies in the literature (Gearhardt et al., 2009a; Brewerton, 2017; Cassin et al., 2019; Munguía et al., 2022). This increased interest in FA can be attributed to several factors, including easy access to calorie-dense foods, the growing obesity epidemic, and advancements in neuroscience, which have prompted researchers to investigate whether addictive mechanisms apply to certain foods. FA is characterized by a hedonic eating behavior involving consuming highly palatable foods in amounts beyond homeostatic requirements (Kalon et al., 2016). Accordingly, studies carried out by Gearhardt et al. (2009a) and Avena et al. (2012) suggest parallels between food-related behaviors and substance use disorders (SUD) due to their impact on the dopaminergic system, among other neuronal pathways (Avena, 2010; Gearhardt et al., 2011). In this context, the “Yale Food Addiction Scale,” developed by Gearhardt et al. (2009b, 2016) assesses food addiction based on SUD criteria in the 5th edition of the Diagnostic and Statistical Manual of Mental Disorders (DSM-5) (Schulte and Gearhardt, 2017). Despite the widespread usage of the term “food addiction” in the literature, the debate over its formal classification as a disorder within the scientific community remains unresolved. Notably, it is not currently recognized as a disease in specialized references such as the DSM-5 Text Revision (DSM-5-TR). It is important to revisit and re-discuss the use of the term “food addiction” to prevent potential stigmatization. Additionally, ongoing debates persist regarding the similarities and differences between FA and other eating disorders (Hauck et al., 2020). Furthermore, the precise mechanisms underlying addictive behaviors related to food have not been fully elucidated.

This Research Topic, “*Neurobiology of food addiction*” aimed to bring up different aspects that guide the neurobiology and behavior related to food addiction, to help to go deeper in the understanding of the topic, considering the substantial gaps in the existing literature.

One important topic highlighted in this editorial is the genetic and epigenetic alterations regarding eating disorders. Sena et al. examined two genetically similar substrains of BALB/c mice to investigate escalated food consumption, compulsive-like food consumption in an aversive context, and incubation of craving after a no-food training period. They found that both substrains of mice showed comparable levels of acute and escalated consumption of palatable food across training trials. Surprisingly, BALB/cByJ mice also revealed binge-like eating even with the unsweetened chow pellets. Respectively, Hidalgo Vira et al. explored genetic variants (rs1799732 and rs1800497) of the dopaminergic pathway previously related to addiction and the potential association with food reinforcement and food addiction in Chilean university students. They observed an association between these variants with anthropometric measurements but addictive behavior did not display a similar association with the studied polymorphisms. This apparent contradiction with previous studies, where a connection between these genetic variants and addictive behaviors related to food had been found, highlights the importance of further investigating the genetic background across different populations.

Different brain regions and multiple factors are involved with the development of reward and motivated-driven behaviors. Among these factors, cues-related rewards stand out as triggers for such behaviors. Two of our articles investigated cue-guided decision-making in rats and humans. In their research, Ghobadi-Azbari et al. studied the neural dynamics underlying food cue-reactivity in overweight/obese participants who reported frequent food cravings. The authors used brain imaging techniques to highlight the involvement of the ventral tegmental area (VTA), amygdala, and orbitofrontal cortex (OFC) in processing food cues. Through dynamic causal modeling (DCM), they probed the effective connectivity between these regions. They found that the VTA, amygdala, and OFC are involved in food cue-reactivity. In particular, the VTA, which has excitatory dopaminergic connections to other regions of the reward network, played a central role in salient cue processing and exerted a strong influence on the amygdala and OFC. Notably, these brain regions played a pivotal role in processing cues related to palatable foods, which may contribute to the reinforcement of addictive eating habits. Yang et al. in turn, investigated dopamine receptor activity in rats and its influence on risky decision-making (RDM). They revealed that dopamine D1 (D1R) and D2 receptors (D2R) in the prefrontal cortex (PFC) played distinct roles in regulating risky choices. The activation of dopamine D1R or the inhibition of D2R on the PFC increased the choice of rats for the risk arm, while inhibition of D1R reduced the choice of rats for this risk. These investigations collectively offer insights into the intricate interplay of brain regions and factors underlying cue-guided behaviors and decision-making processes, shedding light on mechanisms potentially linked to addictive eating habits and risky choices.

Another less-discussed factor impacting reward-seeking and decision-making is sensory stimulus. The olfactory system, intricately intertwined with the brain's reward and appetite regulation centers, emerges as a pivotal factor in how we perceive and interact with food. He et al. utilizing *Drosophila melanogaster* as a model system, studied the role of olfactory sensory neurons (OSNs) on food intake, metabolic aspects, and stress responses. In a well-conducted study, they showed that OSN dysfunction decreased food intake, increased resistance to starvation by modulating lipid metabolism, and reduced cold stress. Their data suggest that these results are due to altered neuropeptide F receptor (NPFR) levels and increased insulin activity in OSNs. This interplay has notable repercussions on metabolic parameters and stress resistance and consequently highlights that the olfactory system is crucial to adapting to the external environment.

Overall, the multidimensional exploration of food addiction through genetic, neural, and sensory lenses provides valuable insights into the intricate mechanisms underlying addictive behaviors. This Research Topic not only deepens our understanding of the complexities of food addiction but also sheds light on the broader field of addictive behaviors, offering potential avenues for future studies and prevention and intervention strategies.

## Author contributions

CL: Writing—original draft. MN: Writing—review & editing.

## References

[B1] Avena N. M. (2010). The study of food addiction using animal models of binge eating. Appetite 55, 734–737. 10.1016/j.appet.2010.09.010 20849896 PMC4354886

[B2] Avena N. M. Gold J. A. Kroll C. Gold M. S. (2012). Further developments in the neurobiology of food and addiction: update on the state of the science. Nutrition 28, 341–343. 10.1016/j.nut.2011.11.002 22305533 PMC3304017

[B3] Brewerton T. D. (2017). Food addiction as a proxy for eating disorder and obesity severity, trauma history, PTSD symptoms, and comorbidity. Eating Weight Dis. Stu. Anorexia Bulim. Obes. 22, 241–247. 10.1007/s40519-016-0355-8 28361213

[B4] Cassin S. E. Buchman D. Z. Leung S. E. Kantarovich K. Hawa A. Carter A.. (2019). Ethical, stigma, and policy implications of food addiction: a scoping review. Nutrients 11, 710. 10.3390/nu11040710 30934743 PMC6521112

[B5] Gearhardt A. N. Corbin W. R. Brownell K. D. (2009a). Food addiction: an examination of the diagnostic criteria for dependence. J. Addict. Med. 3, 1–7. 10.1097/ADM.0b013e318193c993 21768996

[B6] Gearhardt A. N. Corbin W. R. Brownell K. D. (2009b). Preliminary validation of the Yale food addiction scale. Appetite 52, 430–436. 10.1016/j.appet.2008.12.003 19121351

[B7] Gearhardt A. N. Corbin W. R. Brownell K. D. (2016). Development of the yale food addiction scale version 2.0. Psychol. Addict. Behav. 30, 113. 10.1037/adb0000136 26866783

[B8] Gearhardt A. N. Davis C. Kuschner R. Brownell D. K. (2011). The addiction potential of hyperpalatable foods. Curr. Drug Abuse Rev. 4, 140–114. 10.2174/1874473711104030140 21999688

[B9] Hauck C. Cook B. Ellrott T. (2020). Food addiction, eating addiction and eating disorders. Proc. Nutr. Soc. 79, 103–112. 10.1017/S0029665119001162 31744566

[B10] Kalon E. Hong J. Y. Tobin C. Schulte T. (2016). Psychological and neurobiological correlates of food addiction. Int. Rev. Neurobiol. 129, 85–110. 10.1016/bs.irn.2016.06.003 27503449 PMC5608024

[B11] Munguía L. Gaspar-Pérez A. Jiménez-Murcia S. Granero R. Sánchez I. Vintró-Alcaraz C.. (2022). Food addiction in eating disorders: a cluster analysis approach and treatment outcome. Nutrients 14, 1084. 10.3390/nu14051084 35268059 PMC8912776

[B12] Randolph T. G. (1956). The descriptive features of food addiction. Addictive eating and drinking. Q. J. Stu. Alcohol 17, 198–224. 10.15288/qjsa.1956.17.198 13336254

[B13] Schulte E. M. Gearhardt A. N. (2017). Development of the modified Yale food addiction scale version 2.0. Eur. Eating Disorders Rev. 25, 302–308. 10.1002/erv.2515 28370722

